# Proportional Downscaling of Glutamatergic Release Sites by the General Anesthetic Propofol at *Drosophila* Motor Nerve Terminals

**DOI:** 10.1523/ENEURO.0422-19.2020

**Published:** 2020-02-20

**Authors:** Shanker Karunanithi, Drew Cylinder, Deniz Ertekin, Oressia H. Zalucki, Leo Marin, Nickolas A. Lavidis, Harold L. Atwood, Bruno van Swinderen

**Affiliations:** 1 Queensland Brain Institute, The University of Queensland, Brisbane 4072, Queensland, Australia; 2 School of Medical Sciences, The University of New South Wales, Sydney 2052, New South Wales, Australia; 3 School of Biomedical Sciences, The University of Queensland, Brisbane 4072, Queensland, Australia; 4 Department of Physiology, University of Toronto, Toronto, Ontario K1P 1J1, Canada

**Keywords:** Drosophila melanogaster, electrophysiology, general anesthesia, neuromuscular junction, neurotransmission, syntaxin1A

## Abstract

Propofol is the most common general anesthetic used for surgery in humans, yet its complete mechanism of action remains elusive. In addition to potentiating inhibitory synapses in the brain, propofol also impairs excitatory neurotransmission. We use electrophysiological recordings from individual glutamatergic boutons in male and female larval *Drosophila melanogaster* motor nerve terminals to characterize this effect. We recorded from two bouton types, which have distinct presynaptic physiology and different average numbers of release sites or active zones. We show that a clinically relevant dose of propofol (3 μm) impairs neurotransmitter release similarly at both bouton types by decreasing the number of active release sites by half, without affecting release probability. In contrast, an analog of propofol has no effect on glutamate release. Coexpressing a truncated syntaxin1A protein in presynaptic boutons completely blocked this effect of propofol. Overexpressing wild-type syntaxin1A in boutons also conferred a level of resistance by increasing the number of active release sites to a physiological ceiling set by the number of active zones or T-bars, and in this way counteracting the effect of propofol. These results point to the presynaptic release machinery as a target for the general anesthetic. Proportionally equivalent effects of propofol on the number of active release sites across the different bouton types suggests that glutamatergic circuits that involve smaller boutons with fewer release sites may be more vulnerable to the presynaptic effects of the drug.

## Visual Abstract

**Figure:**
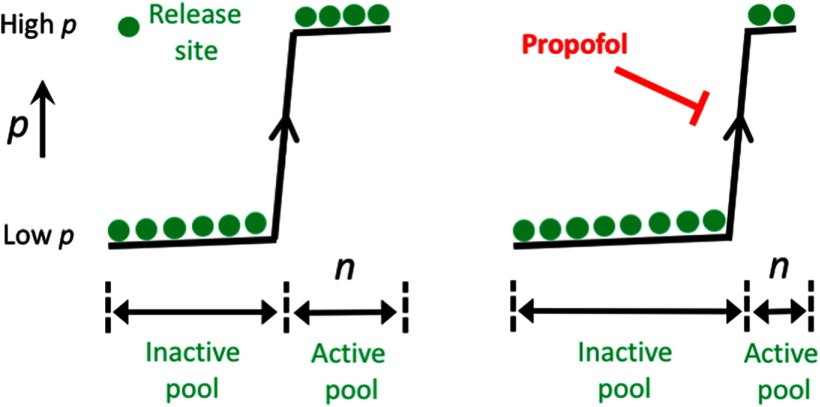

## Significance Statement

Over 200 million surgeries are performed worldwide under general anesthesia every year, and the anesthetic of choice is increasingly the intravenous agent propofol. Sedation produced by propofol is understood to result from postsynaptic activation of inhibitory mechanisms in the brain. We have identified a presynaptic effect of propofol on excitatory synapses. Recording from individual glutamatergic boutons in fly larvae, we found that a clinically relevant dose of propofol impairs glutamate release by proportionally decreasing the participation of release sites across different bouton types. This suggests that propofol anesthesia involves presynaptic as well as postsynaptic mechanisms, and a proportional effect in large and small boutons may explain why some circuits are more vulnerable than others to the effects of the drug.

## Introduction

In the 3 decades since its discovery, propofol has become the most commonly used general anesthetic for surgical procedures in humans. Like many other general anesthetics, propofol is understood to potentiate inhibitory pathways in the brain by acting on postsynaptic GABA_A_ receptor subunits (Franks, 2008). Thus, an increasingly accepted idea for propofol-induced sedation is that it engages endogenous sleep pathways in the brain (Hemmings et al., 2019). However, sleep pathways cannot be the entire explanation, since—unlike sleep—surgical anesthesia is by definition not reversible as long as an adequate dose of the drug is applied. Additionally, there is growing evidence that general anesthesia more broadly is physiologically different from sleep, when investigated at the level of the neural circuitry involved (Brown et al., 2011) and the associated brain activity measures (Akeju and Brown, 2017). This suggests alternate target mechanisms that might complement the better understood processes relating to postsynaptic GABA_A_ receptor function.

Recent work has shown that GABA-acting drugs such as propofol target a number of proteins other than GABA_A_ receptors, including TrpA1 channels (Ton et al., 2017) and kinesin (Woll et al., 2018), resulting in parallel consequences on neural functions alongside sleep induction. Another emerging target mechanism is the presynaptic release machinery (van Swinderen and Kottler, 2014). There is increasing evidence in animal models as well as cell culture preparations that general anesthetics also act presynaptically (van Swinderen et al., 1999; Metz et al., 2007; Herring et al., 2009, 2011; Xie et al., 2013; Baumgart et al., 2015; Zalucki et al., 2015; Troup et al., 2019). Exactly how general anesthetics impair neurotransmission remains unclear, with isoflurane affecting presynaptic sodium and calcium channels (Koyanagi et al., 2019; Zhou et al., 2019) as well as SNARE formation (Xie et al., 2013; Troup et al., 2019); however, it is unknown whether intravenous and volatile agents might have common presynaptic effects. Work in rat neurosecretory PC12 cells showed that clinical concentrations of both propofol and isoflurane inhibit neurotransmission from these cells, and that coexpression of a mutant form of the presynaptic protein syntaxin1A preserves neurotransmission in these cells under anesthetic exposure (Herring et al., 2009, 2011). Syntaxin1A is a key membrane-bound component of the presynaptic release machinery, which is necessary to enable vesicular release of neurotransmitters (Südhof and Rizo, 2011). The effect of general anesthetics on syntaxin1A remained unclear, however, until the advent of super-resolution microscopy techniques that permitted visualizing and tracking single molecules through time (McKinney et al., 2009). In a recent super-resolution imaging study of a similar PC12 cell preparation, we confirmed a presynaptic effect of propofol and showed that the intravenous anesthetic impairs neurotransmission by restricting the mobility syntaxin1A on the cell membrane, resulting in nonfunctional nanoclusters (Bademosi et al., 2018). The same syntaxin1A clustering effect was also found in a live animal preparation, namely motonerve terminals of the fly larval neuromuscular junction (NMJ; Bademosi et al., 2018), suggesting a common presynaptic target mechanism across different cell types and animal species. Together, these results also suggested an anesthetic effect more proximal to the presynaptic release machinery, at least for some intravenous agents such as propofol. One compelling hypothesis emerging from these findings is that propofol-impaired neurotransmitter release could result from decreased availability of syntaxin1A at release sites on the plasma membrane. Release sites, also referred to as “active zones,” are where the presynaptic machinery comes together to facilitate the fusion of synaptic vesicles with the cell membrane (Atwood, 2006; Neher and Brose, 2018). Although multiple studies have now shown that general anesthetics impair neurotransmission, what remains unclear is how exocytosis physiology is affected in “real” synapses, in live animals. In the current study, we return to the fly larval NMJ to test how a clinically relevant dose (3 μm) of propofol impairs glutamate release from individual motonerve terminals, or boutons. We further assess how coexpression of mutant and wild-type syntaxin1A protein modulate these presynaptic effects.

## Materials and Methods

### Fly stocks

*Drosophila melanogaster* strains were maintained on standard yeast-based medium at 22° on a 12 h light/dark cycle. Flies were grown at low density to promote larval health. Food was kept moist with drops of additional distilled water as required, to promote burrowing in developing larvae. Strains used in this study were wild-type Canton S (CS), *Elav-Gal4*, *UAS-syx-227* (Troup et al., 2019), *UAS-syx-FL* (Troup et al., 2019). Transgenic strains were outcrossed to a w^1118^ wild-type background. To generate syntaxin1A mutant or wild-type overexpression animals, homozygous UAS-syx-227 or UAS-syx-FL males were crossed to homozygous elav-Gal4 female virgins.

### Preparation of larvae for electrophysiological recordings

Electrophysiological experiments were performed on muscle 6, abdominal segment 3, at room temperature (22°C) in wandering third instar larvae of either sex. This muscle is innervated by 1b and 1s motoneurons, which are functionally different. Experiments were conducted in HL3 hemolymph-like physiological solution of the following composition (in mm): 70 NaCl, 5 KCl, 1 CaCl_2_, 20 MgCl_2_, 10 NaHCO_3_, 5 trehalose, 115 sucrose, and 5 BES [(*N*,*N*-bis(2-hydroxyethyl)-2-aminoethane sulfonic acid)] or HEPES, pH 7.2 and ∼340 mOsm. The 1.5 mm CaCl_2_ was used for some experiments. Karunanithi et al. (2018) provides detailed descriptions of the preparation of the larvae for electrophysiological recordings, the focal macropatch recording technique used for recording postsynaptic currents from single boutons, the assessment of the quality postsynaptic current recordings, the analysis and interpretation of the results, and the method for perfusing drugs into the recording chamber. Data from both sexes was combined.

### Focal macropatch recordings from single boutons: rationale

The most explicit requirement for conducting proper quantal analysis is that the evoked and spontaneous postsynaptic responses be recorded from the synapses formed by a single input neuron (Del Castillo and Katz, 1954); and ideally at a single release site (Bekkers, 1994). Adhering to that requirement enables proper estimates of quantal parameters, namely quantal content (QC), quantal size, release probability, and active release site numbers, that being irrespective of the method used, assuming that that method adheres to requirements for proper quantal analysis (McLachlan, 1978; Korn and Faber, 1991; Clements, 2003). Meeting that requirement is not straightforward in larval muscles. This is because the larval muscles are multiply innervated (Hoang and Chiba, 2001). Virtually all larval muscles receive synaptic inputs from the highly active 1b and the less active 1s motoneurons (Chouhan et al., 2010; Newman et al., 2017). The 1b and 1s motor nerve terminals are composed of boutons, which form synapses with the muscle (Atwood et al., 1993). The 1b boutons form more synapses than 1s boutons with the muscle (Atwood et al., 1993). Also, compared with 1s boutons, 1b boutons exhibit smaller evoked and spontaneous mean postsynaptic response amplitudes, smaller release probabilities (Karunanithi et al., 2002; Pawlu et al., 2004; Dawson-Scully et al., 2007; Newman et al., 2017; Li et al., 2018), but higher active release site numbers (this article). Finally, 1b boutons are facilitating, whereas 1s boutons are depressing (Kurdyak et al., 1994; Newman et al., 2017). Considering the significant difference between 1b and 1s synaptic properties, commonly used recording methods at the larval NMJ, namely, intracellular recordings and two-electrode voltage clamping (TEVC) cannot discriminate between the 1b and 1s postsynaptic responses, and will yield inaccurate estimates of quantal parameters.

To circumvent such problems, we used the focal macropatch recording technique (FMRT) to unambiguously record postsynaptic currents from individual 1b and 1s boutons (Karunanithi et al., 2002, 2018; Pawlu et al., 2004; Kittel et al., 2006; Dawson-Scully et al., 2007). Like any technique, it needs to be performed correctly to get the proper results. In the case of FMRT, focal macropatch electrodes need to be fabricated in a manner to enable it to sit flush on the surface of the muscle fiber, without damaging it, when recording postsynaptic currents from the enclosed bouton (Karunanithi et al., 2018). The smaller evoked [excitatory junctional current (EJC)] and spontaneous [miniature EJC (mEJC)] mean amplitudes at 1b boutons than at 1s boutons were first observed using this technique (Karunanithi et al., 2002; Pawlu et al., 2004; Dawson-Scully et al., 2007) and were subsequently confirmed using optical quantal analysis (Newman et al., 2017; Li et al., 2018). Using FMRT, it was also shown that quantal amplitudes varied linearly with vesicle sizes, indicating that quantal amplitudes clearly reflected the transmitter content of individual synaptic vesicles and that postsynaptic receptors were unsaturated by a quantum of neurotransmitter (Karunanithi et al., 2002). Clear resolution of quantal amplitudes and of their variation at a selected synapse type is a prerequisite for quantal analysis (Korn and Faber, 1991), and those findings added confidence in using FMRT. The large signal-to-noise ratios observed in recordings of quantal currents indicate that no additional signal processing is required to remove contaminating noise from the signals during analysis, greatly simplifying analysis (Karunanithi et al., 2002, 2018). Mostly, a single vesicle is released from a single release site at low-frequency nerve stimulation (Melom et al., 2013), and the released neurotransmitter does not saturate postsynaptic receptors (Karunanithi et al., 2002). The evoked postsynaptic responses from single boutons are therefore not saturated responses, but proportional to the number of vesicles released from active release sites (Melom et al., 2013).

At larval NMJs, optical quantal analysis holds great promise to enable quantal analysis at not only single-bouton resolution, but also at single-release site resolution (Melom et al., 2013; Newman et al., 2017; Li et al., 2018). However, we chose not to use optical analysis because of the following drawbacks. (1) The responses are extremely protracted in time course, lasting hundreds of milliseconds and being 10–20 times the time course of quantal currents recorded using TEVC or FMRT (Bademosi et al., 2018; Li et al., 2018). Such responses are unphysiological since they do not reflect the speed of responses, which are characteristic of glutamatergic synapses. Such protracted time courses are also detrimental toward studying mechanisms of short-term presynaptic plasticity, which operate at submillisecond-to-millisecond timescales (Regehr, 2012; Kaeser and Regehr, 2017). (2) Estimates of quantal parameters using optical analysis at single boutons could be inaccurate if inadequate numbers (<10) of events are collected. Normally, adequate numbers of evoked (150–300 events) and spontaneous (25–50 events) postsynaptic responses are needed to correctly account for the variation in response amplitudes to conduct proper quantal analysis. Since the frequency of spontaneous events occurring at single larval NMJ boutons is very low, ∼0.04 Hz (Karunanithi et al., 1999; Bademosi et al., 2018), it would take ∼10 min to collect a minimum of 25 events. Continuous imaging for 10 min would likely bleach the preparation, resulting in misestimates of quantal parameters. (3) There has been no correlation made for optical imaging to establish that variations in quantal amplitude truly reflect the variations in released neurotransmitter from individual vesicles. In that regard, FMRT offers significant advantages for our analysis in making proper estimates of quantal parameters at the level of single synaptic boutons.

### Focal macropatch recordings from single boutons: methodology

A brief account of the methods for conducting focal macropatch recordings are provided as follows. The preparation was viewed with a 60× water-immersion lens [numerical aperture (NA), 1.0] using Nomarski optics. Live images of the nerve terminals were captured using a low-light video camera and projected onto a computer monitor. Such a setup enabled the selection of well isolated, single 1b and 1s boutons for focal macropatch recordings and the positioning of the focal macropatch electrode at the site of recording. The 1s and 1b boutons were identified under Nomarski optics (Kurdyak et al., 1994; Karunanithi et al., 2018). The 1b nerve terminals are composed of larger and more variable-sized boutons (3–5 μm in diameter) than are 1s nerve terminals (boutons, ∼3 μm in diameter; Atwood et al., 1993; Kurdyak et al., 1994). Well isolated 1s or 1b boutons were selected for recordings. The focal macropatch electrodes possessed open tip diameters of approximately ∼5 μm and were filled with HL3 solution. The open tip does not exert direct pressure onto the bouton during recordings (Karunanithi et al., 2002). The recorded signals were amplified using an Axoclamp 900A amplifier (under bridge/I-clamp mode; Molecular Devices), digitized (at an acquisition rate of 40 kHz), and stored on a computer using the PowerLab 4/35 data acquisition system (ADInstruments). In each experiment, focal macropatch recordings were made from a single 1b or a single 1s bouton, or from two boutons of each type. In each experiment, 200 EJCs and 25–30 mEJCs were recorded from a single bouton. The stimulating electrode (lumen diameter, ∼10–12 μm), filled with perfusate, was used to gently suck the cut end of the segmental nerve and was stimulated at a frequency of 1 Hz at supramaximal strengths to evoke EJCs.

The measured EJC and mEJC amplitudes were used to derive quantal size (*q*; average mEJC amplitude), average EJC amplitude, QC (average EJC amplitude/average mEJC amplitude), and the quanta released per stimulus. In each experiment, the quanta released per stimulus were calculated by dividing each EJC amplitude in the train by the average mEJC amplitude (Bademosi et al., 2018). The cumulative relative frequency histogram for each group represents the pooled data from all the experiments in that group. For each experiment, QC should equal the average quanta released per stimulus.

At high [Ca^2+^]_o_, neurotransmitter release can be described by a binomial model, where QC = *np* (Del Castillo and Katz, 1954; McLachlan, 1978). *n* equates to the average number of functionally active release units/sites, and *p* equates to the average release probability of those active sites (Lavidis, 1995). In each experiment, *p* was calculated using the following equation:

$$p=1-\frac{S^{2}}{QCq^{2}}+\frac{\sigma^{2}}{q^{2}}.$$

where *q* is the quantal size, *S^2^* is the variance of the EJC amplitudes, and σ^2^ is the variance of the mEJC amplitudes (Bennett and Florin, 1974; McLachlan, 1978; Lavidis and Bennett, 1992). *n* was calculated by dividing QC by *p*.

For measuring quantal release kinetics (mEJC rise times and decay constants), we used LabChart Pro software (version 8.1.5; ADInstruments). The mEJC decay time constant was measured by fitting a single exponential to the mEJC decay, using the Peak Analysis subroutine. Fits of decay were manually checked for accuracy. Rise times were measured manually, using the two cursors placed at the base and the peak of each mEJC, as done previously (Karunanithi et al., 1999).

### Drug perfusion

A syringe pump (KDS 100 Legacy Syringe Pump, KD Scientific) was used to perfuse the preparation with perfusate throughout all experiments (Zalucki et al., 2015). Perfusion was conducted at rates of 1–2 ml/min over 30–40 min before recordings were performed and also throughout the recording session. In each experiment, recordings were brief, taking ∼3.5 min for EJCs and 5–10 min for mEJCS. When drugs were to be perfused into the bath, stock concentrations of propofol and the propofol analog 2,4-diisopropylphenol (Sigma-Aldrich) were prepared in DMSO at 0.4% v/v concentration and then dissolved into the HL3 solution. To ensure that perfusate had equilibrated in the animal tissue, preparations were exposed to propofol-containing perfusate (or analog-containing solutions) before recording electrode placement. Similarly, in control experiments, the preparations were perfused with the perfusate (lacking propofol) before recording electrode placement. The focal macropatch electrode was also left immersed in the perfusate before the recording session, ensuring that the drug concentration within the lumen of the electrode equilibrated to that outside of it. By taking such measures and by considering that the period of recording was brief relative to the period of perfusion, the drug concentration within the lumen of the electrode could be considered as being stable. Similar procedures were undertaken in control experiments; however, the perfusate lacked drugs. Drug concentrations were in a clinically relevant range (3 μm), as determined previously (Bademosi et al., 2018). Higher propofol concentrations (30 μm) led to a 50% failure rate per stimulus in this preparation (compared with 0% failure rate for 3 μm), suggesting that at supraclinical drug concentrations, mechanisms other than just neurotransmitter release were also being compromised, such as failures of nerve action potentials to invade the motor nerve terminals and the attenuation of nerve-evoked calcium influx. We therefore restricted our quantal analysis in this study to 3 μm propofol, which was unaffected by action potential failures.

### Serial electron microscopy used to determine T-bars per bouton

Experiments and analyses were performed as previously described (Karunanithi et al., 2002) to determine the number of T-bars/active zones per bouton on muscle 6, segment A3 in CS larvae. Three larvae were serially sectioned and used for analysis. The thickness of each section was 75 nm. A series of thin sections were cut along muscle 6, and measurements of T-bar numbers per bouton made from 1b and 1s boutons within those series. Electron micrographs along a series were used to serially reconstruct the string of boutons, along with some of their key structural features. A bouton was identified as a structure whose cross-sectional area changed approximately fivefold at the beginning and at the end from its mid-cross-sectional area (Lnenicka et al., 1986). Within each bouton, synapses were identified as those electron-dense presynaptic and postsynaptic membranes in close apposition, and T-bars were those dense projections located on the presynaptic membrane, surrounded by a cloud of synaptic vesicles. We determined the number of T-bars per bouton by counting the number of T-bars located on synapses within each bouton.

### Immunochemistry

Wandering third instar larvae were dissected in fresh extracellular fluid and fixed for 20 min with cold 4% paraformaldehyde in 1× PBS. The preparation was blocked with 0.2% Triton X-100, 2% bovine serum albumin (Jackson ImmunoResearch), or 10% normal goat serum (Sigma-Aldrich) in PBS-T (1× PBS, 0.2 Triton X-100) for 30 min. The following antibodies were used: HRP-Cy3 (1:120; Jackson ImmunoResearch), Bruchpilot (mouse anti-nc82, 1:100; Developmental Studies Hybridoma Bank), and HA (rabbit anti-HA, 1:1000; Cell Signaling Technology). Primary antibody incubations were performed at room temperature for 1 h. Secondary antibodies were diluted in blocking buffer in PBS-T [Invitrogen Alexa Fluor 488 goat anti-mouse (1:250) and Alexa Fluor 647 goat anti-rabbit, Thermo Fisher Scientific]. Samples were incubated with secondary antibodies for 3 h. The preparations were mounted in Vectashield (Vector Laboratories). Imaging of the nerve terminals innervating muscle 6 was performed on a Zeiss 710 Meta confocal microscope and on a spinning-disk confocal system (Marianas, 3I) consisting of an Axio Observer Z1 (Carl Zeiss) equipped with a CSU-W1 spinning-disk head (Yokogawa Corporation of America), ORCA-Flash4.0 version 2 sCMOS camera (Hamamatsu Photonics) using a 63× 1.4 NA PlanApo oil-immersion objective. Mouse HA-monoclonal antibody (1:3000) was used to tag HA-Syntaxin1A and HA-Syntaxin1A^227^. The secondary antibody was donkey anti-mouse Invitrogen Alexa Fluor 647 (1:500; Thermo Fisher Scientific).

### Quantification of active zone number

Images were processed on Fiji (ImageJ) software. Approximately 20 1s and 1b boutons were selected per animal for quantification, 10 per side. Two blinded experimenters counted the number of puncta at the selected terminals independently. Representative images were deconvolved by using Huygens Pro software (Scientific Volume Imaging).

### Experimental design and statistical analysis

Statistical and data analysis was performed using Excel (Microsoft) and Prism 6 (GraphPad Software) software. To assess statistical differences between two groups, the unpaired (two-tailed) Student’s *t* test was used, with Welch’s correction. The nonparametric tests, one-way ANOVA and the Kolmogorov–Smirnov (K–S) test were used to assess statistical differences. In all cases, significance was set at *p* < 0.05. The K–S test was used to compare the shapes of standardized distributions. Distributions were standardized by subtracting the average and then normalizing to 1 SD. In rank-ordered plots, values were rank ordered in ascending order and then plotted against one another (Turrigiano et al., 1998). The coefficient of variation was obtained by dividing the SD of a population by its average. Where possible, normalized parameters were provided in the figures to improve readability, with the non-normalized data provided as Extended Data. Parameters were normalized to their respective averages in controls. All data are available in the main text, figures, or the Extended Data. Raw data, code, and materials used in the analysis are available on request from the corresponding author.

## Results

### Propofol decreases glutamate release similarly at different bouton types

**Figure 1.**
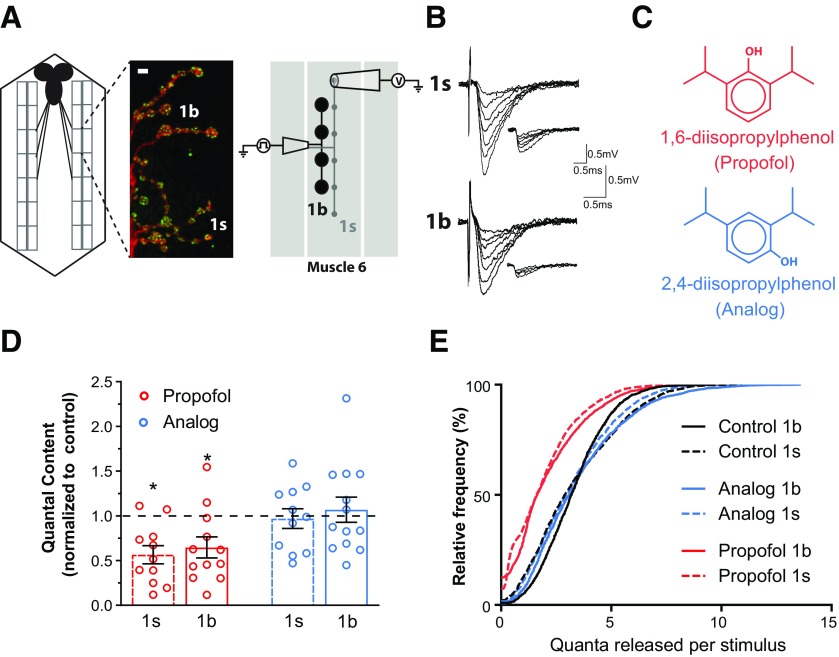
Propofol decreases glutamate release similarly from different synapse types. ***A***, Left, Schema of *Drosophila* larval NMJ preparation. The black structure is the brain, rectangles are muscle segments. Middle, Muscle 6 motor nerve terminals labeled with HRP to identify neurons and nc82 to identify active zones. 1s and 1b boutons are indicated. Scale bar, 4 μm. Right, Diagram illustrating the configuration for focal macropatch recording from single 1s and 1b boutons at the fly larval NMJ. ***B***, Example traces of EJCs and mEJCs (inset) recorded from 1s and 1b boutons in the CS strain. ***C***, Chemical structures of propofol (1,6-diisopropylphenol) and its nonanesthetic analog, 2,4-diisopropylphenol. ***D***, Normalized QC in drug-treated preparations (3 μm) for both bouton types. Parameters were normalized to their respective averages in non-drug-treated controls (dashed line). Raw data are presented in Extended Data Figure 1-1. *p <* *0.05; Student’s *t* test. Graphs show individual data points (animals), and error bars show the average ± SEM. ***E***, Cumulative plot of quanta released per stimulus, for both bouton types and both drugs.

To measure propofol effects on neurotransmitter release, we used electrophysiological recordings from individual glutamatergic boutons in *Drosophila* larvae (Karunanithi et al., 2018; Fig. 1*A*). The *Drosophila* larval NMJ is a highly studied model glutamatergic synapse that shares similarities with mammalian glutamatergic synapses (Menon et al., 2013; Harris and Littleton, 2015). Fly larval NMJs are unusual in that virtually all the muscles receive inputs from two distinct motoneurons (Hoang and Chiba, 2001), as follows: the larger, highly active 1b and the smaller, less active 1s motoneurons (Chouhan et al., 2010; Newman et al., 2017; Fig. 1*A*). The nerve terminals of 1s and 1b motoneurons are composed of synaptic boutons, which form synapses with the muscle (Atwood et al., 1993; Newman et al., 2017). Nearly all nerve terminals on the presynaptic side possess release sites (or active zones) where neurotransmitter release occurs, and 1b boutons possess on average more release sites than 1s boutons (Atwood et al., 1993; Kittel et al., 2006; Wagh et al., 2006). Despite this, 1b boutons have lower release probabilities and generate smaller postsynaptic responses than 1s boutons (Kurdyak et al., 1994; Newman et al., 2017). The 1s and 1b synapses are therefore referred to as “strong” and “weak,” respectively (Kurdyak et al., 1994; Atwood and Karunanithi, 2002; Newman et al., 2017).

10.1523/ENEURO.0422-19.2020.f1-1 Figure 1-1 ***A–C***, Raw data of EJC amplitude (***A***), mEJC amplitude (*B*), and QC (C) used to generate the normalized data in Figure 1*D*. *p <* *0.05; **p <* *0.01; ***p <* *0.001; #*p *<* *0.0001; Student’s *t* test. ***D***, 1b and 1s mEJC frequencies recorded from single boutons in control and in drug-treated preparations. ***E***, An exemplary mEJC trace (red) showing the measure of rise time and the single exponential fit to the decay phase (black) from which the decay constant is derived. ***F***, ***G***, mEJC rise time (***F***) and decay constant (***G***). Significant differences are shown: *p <* *0.05; **p < 0.01; Student’s *t* test. Graphs show individual data points and error bars show average ± SEM. Download Figure 1-1, EPS file.

We distinguished between the effects of propofol on 1b and 1s neurotransmitter release by recording postsynaptic currents from single 1b and 1s boutons, using focal macropatch electrodes (Karunanithi et al., 2018; Fig. 1*A*; for details on why this recording preparation is appropriate for the questions posed here, see Materials and Methods). We recorded the stimulus-evoked EJCs and the mEJCs that resulted when individual vesicles spontaneously released neurotransmitter (Fig. 1*B*). From measures of the mean amplitudes of EJCs (estimating synaptic strength) and mEJCs (estimating quantal size) in each experiment, we determined the amount of neurotransmitter released by deriving QC (average number of vesicles released per stimulus; Karunanithi et al., 2018). These results confirmed that propofol (Fig. 1*C*) decreased QC at both bouton types (Fig. 1*D*,*E* and Extended Data Fig. 1-1*A–C*; Bademosi et al., 2018). In contrast, an analog of propofol, 2, 4-diisopropylphenol (Fig. 1*C*), had no effects on QC (Fig. 1*D*,*E* and Extended Data Fig. 1-1*A–C*). A more detailed examination of the number of quanta released per stimulus showed a consistent decrease under propofol but no change for the analog, compared with controls (Fig. 1*E*). In conclusion, glutamate release appears similarly impaired by propofol at 1b and 1s synapses, while the analog does not appear to affect glutamatergic neurotransmission in this preparation.

We further tested whether propofol affected mEJC frequency and postsynaptic receptor kinetics. 1b and 1s mEJC frequencies were unaffected by either propofol or its analog (Extended Data Fig. 1-1*D*), indicating that propofol did not affect spontaneous vesicle fusion. At mammalian inhibitory synapses, propofol prolongs the decay of inhibitory postsynaptic currents (Kitamura et al., 2003). To determine whether propofol was acting directly on postsynaptic current kinetics, we analyzed mEJC rise and decay times (Extended Data Fig. 1-1*E*). Propofol did not affect either the averaged rise times or the decay time constants at either bouton type (Extended Data Fig. 1-1*F*,*G*). The analog did, however, modestly prolong both those parameters at 1b boutons (Extended Data Fig. 1-1*F*,*G*).

### Determining the number of active release sites in 1s and 1b boutons

**Figure 2.**
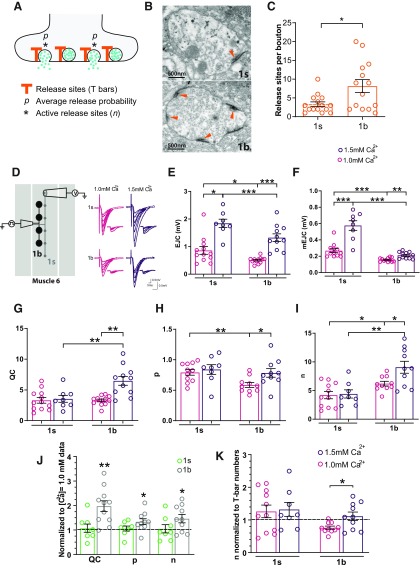
The upper limit for *n* is set by the number of T-bars. ***A***, Schema of release sites at a synapse. Active release sites (*) contribute to the average release *p*. ***B***, Electron micrographs (EMs) of a 1s (top) and a 1b (bottom) bouton, with T-bars (synapses) indicated with arrowheads. ***C***, Average number (±SEM) of release sites for 1s and 1b boutons, determined from the T-bar counts from the EM data. See Materials and Methods, and Extended Data Figure 2-1 for other examples. ***D***, [Ca^2+^]_e_ was raised from 1.0 to 1.5 mm in focal macropatch recordings in CS larvae. Right, Sample recordings from single 1s and 1b boutons revealed increases in the amplitudes of EJCs and mEJCs at both bouton types under higher calcium concentrations. ***E***, Average EJC (±SEM) for 1s and 1b recordings. ***F***, Average mEJC (±SEM) for 1s and 1b recordings. ***G***, As [Ca^2+^]_e_ was raised, calculations of QCs revealed no increase in 1s QC, whereas there was an increase in 1b QC. ***H***, ***I***, There were no increases in *p* (*H*) or in *n* (*I*) at 1s boutons, whereas there were increases in both *p* (***H***) and *n* (***I***) at 1b boutons, contributing to the increase in 1b QC (***G***). ***J***, We observed that at 1s boutons, all the release sites were already activated at 1.0 mm [Ca^2+^]_e_, with no additional increase being observed when [Ca^2+^]_e_ was further raised. At 1b boutons, not all of the release sites were activated at 1.0 mm [Ca^2+^]_e_, whereas they were all activated when [Ca^2+^]_e_ was further raised. ***K***, We generated a graph to test whether values of *n* approached the number of T-bars per bouton as [Ca^2+^]_e_ was raised, by normalizing *n* (Fig. 2*I*) to the number of T-bars per bouton (Fig. 2*C*). The dashed line at 1.0 is representative of when *n* equates to the number of T-bars per bouton. These results indicate that *n* is limited by the number of T-bars per bouton type. *p <* *0.05; **p <* *0.01; ***p <* *0.001; Student’s *t* test. 1.0 mm [Ca^2+^]_e_ data are the same as in Figure 1, shown here for comparison. Graphs show individual data points (animals), and all error bars are ±SEM.

We next investigated how propofol decreased QC at individual boutons. This first required estimating two key aspects of QC, namely the number of active release sites per bouton, and their associated release probability. At high [Ca^2+^]_e_ (1 mm), which is close to physiological concentrations (Atwood and Karunanithi, 2002), QC is described by the equation QC = *np*, where *n* represents the average number of functionally active release sites, and *p* represents the average release probability (average chance of a vesicle releasing neurotransmitter at an active release site, following a stimulus; Del Castillo and Katz, 1954; McLachlan, 1978; see Materials and Methods). Stimulation at low frequencies, as used here, causes neurotransmitter to be released from a single vesicle at a single release site (Melom et al., 2013). The model of quantal release from a bouton can thus be visualized as vesicles releasing neurotransmitter from a population of *n* active release sites with an average probability, *p* (McLachlan, 1978; Fig. 2*A*). Within boutons, the anatomic correlates of release sites are the T-bars (Kittel et al., 2006; Wagh et al., 2006; Fig. 2*B* and Extended Data Fig. 2-1). We found that the upper limit for the number of active release sites per bouton was set by the number of T-bars, which were approximately four in 1s boutons and eight in 1b boutons, although there was considerable variability within bouton types (Fig. 2*B*,*C* and Extended Data Fig. 2-1). To ascertain that our physiological estimates of *n* conformed with the actual physical number of active zones per bouton, we recorded from 1s and 1b boutons under increased calcium concentrations (1.5 mm) to maximize neurotransmission (Fig. 2*D*), and calculated *n* and *p* for these data, compared with physiological (1.0 mm) data. Increasing the calcium concentration increased EJC and mEJC amplitudes in both 1s and 1b recordings (Fig. 2*E*,*F*), although quantal content was only increased in 1b (Fig. 2*G*). Release probability (*p*) and the number of active release sites (*n*) remained unchanged in 1s, whereas both increased in 1b (Fig. 2*H*,*I*). Increasing calcium thus had no effect on 1s boutons, but significantly increased the number of active release sites in 1b, and therefore quantal content (Fig. 2*J*). Importantly, raising calcium to 1.5 mm increased *n* to the physical ceiling set by the average number of T-bars expected in 1b boutons (approximately eight), whereas *n* remained at the ceiling already achieved in 1s boutons (approximately four) at physiological calcium concentrations (Fig. 2*K*). This shows that, at calcium concentrations of 1.0 mm, all the release sites are active in 1s boutons, whereas only a fraction are active in 1b boutons, but when the calcium concentration was raised further to 1.5 mm, all the release sites in both 1s and 1b boutons became active. This also shows that our physiological calculations of *n* match well with the actual anatomic number of release sites observed in either bouton type. Our accurate assessment of *n* allowed us to then evaluate the effect of propofol on glutamate release at physiological [Ca^2+^]_e_: is propofol decreasing QC by decreasing release *p*, the *n* of active release sites, or both?

10.1523/ENEURO.0422-19.2020.f2-1 Figure 2-1 Example electron micrographs of 1s and 1b boutons, with close-ups of active zones. 1s and 1b boutons are distinguished by the number of active zones (T-bars, arrowheads), although this can be highly variable. Other distinguishing features are larger vesicle sizes in 1s boutons and more subsynaptic reticulum structures in 1b boutons. Download Figure 2-1, EPS file.

### Propofol decreases the number of active release sites in 1s and 1b boutons

**Figure 3.**
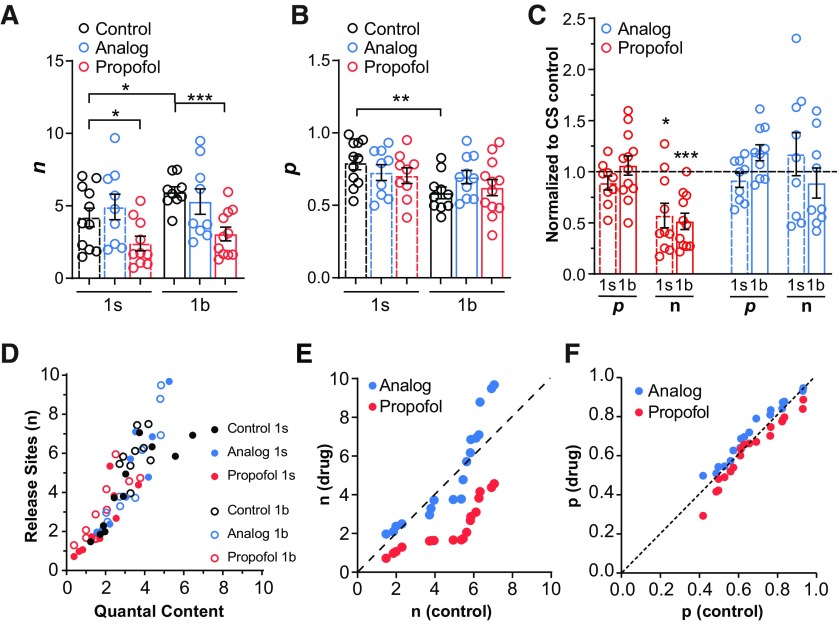
Propofol decreases the number of active release sites at synapses. ***A***, Estimated number of active release sites (*n*; see Materials and Methods) in 1s and 1b boutons, for control and drug-treated conditions, in CS animals. ***B***, Estimated release probability (*p*, see “Materials and Methods”) in 1s and 1b boutons, for control and drug-treated conditions, in CS animals. ***C***, Data from ***A*** and ***B***, normalized to non-drug-treated controls (dashed line). Graphs in ***A–C*** are averages ± SEM; individual data points (animals) are shown. *p <* *0.05; **p <* *0.01; ***p <* *0.05, Student’s *t* test. ***D***, Quantal content plotted against *n*, from all 1s and 1b recordings in control and drug conditions. ***E***, Rank-ordered plot of *n* for drug versus control conditions. Dashed line indicates no effect of drug on *n*. ***F***, Rank-ordered plot of *p* for drug versus control conditions. Dashed line indicates no effect of drug on *p*.

Interestingly, propofol had the exact same effect at 1s and 1b boutons: it lowered QC by decreasing *n* and not *p* at both bouton types, whereas the analog, as expected, had no effects on these parameters (Fig. 3*A*,*B*). Normalizing the data to controls revealed that propofol had a proportionally equivalent effect on 1b and 1s boutons (Fig. 3*C*), decreasing the number of functionally active release sites (*n*) by half, without affecting the release probability (*p*). The fact that *p* did not change under propofol exposure indicates that the remaining active release sites were not compromised. These uncompromised release sites probably contributed to the large variation in QC observed among preparations (Fig. 1*D*). If more release sites were compromised by propofol, the variations in QC would have been smaller, since neurotransmitter release would have been muted at some boutons.

We next plotted the relationship between *n* and QC across all of our experiments (Fig. 3*D*) to better understand how *n* was distributed among our data. Although 1s and 1b boutons have on average a different number of active zones (Fig. 2*C* and Extended Data Fig. 2-1), we observed a linear relationship between *n* and QC across all tested conditions when data from 1s and 1b boutons were combined (Fig. 3*D*). This showed considerable overlap between bouton types, suggesting that knowing the baseline *n* is more important for detecting the effect of the drug, rather than knowing what the bouton type was. In other words, the number of active release sites is highly variable per bouton, and the difference between 1s and 1b boutons is hardly discrete in this regard. We therefore next examined whether boutons possessing specific numbers of functionally active release sites were more affected than others by propofol, irrespective of bouton type. To do this, we rank ordered (in ascending order) *n* derived from control and from propofol-treated preparations and plotted them against one another (Turrigiano et al., 1998; Fig. 3*E*). Points clustered close to the dashed line represent cases where there were no or minimal effects of propofol. Consistent with our previous analysis (Fig. 3*C*), we found that propofol decreased the number of active release sites by about half for most values of *n*, with the strongest effects observed in boutons displaying an *n* value between 4 and 6 (Fig. 3*E*). As anticipated, we did not observe any such effects with the analog (Fig. 3*E*). As neither propofol nor its analog had an effect on release probability, we observed all points clustered on the dashed line in rank-ordered plots for *p* (Fig. 3*F*). These results indicate that the effect of propofol on neurotransmission is linked to the number of functionally active release sites available, irrespective of bouton type.

### Syntaxin1A manipulations confer resistance to propofol by preserving or increasing the number of active release sites

**Figure 4.**
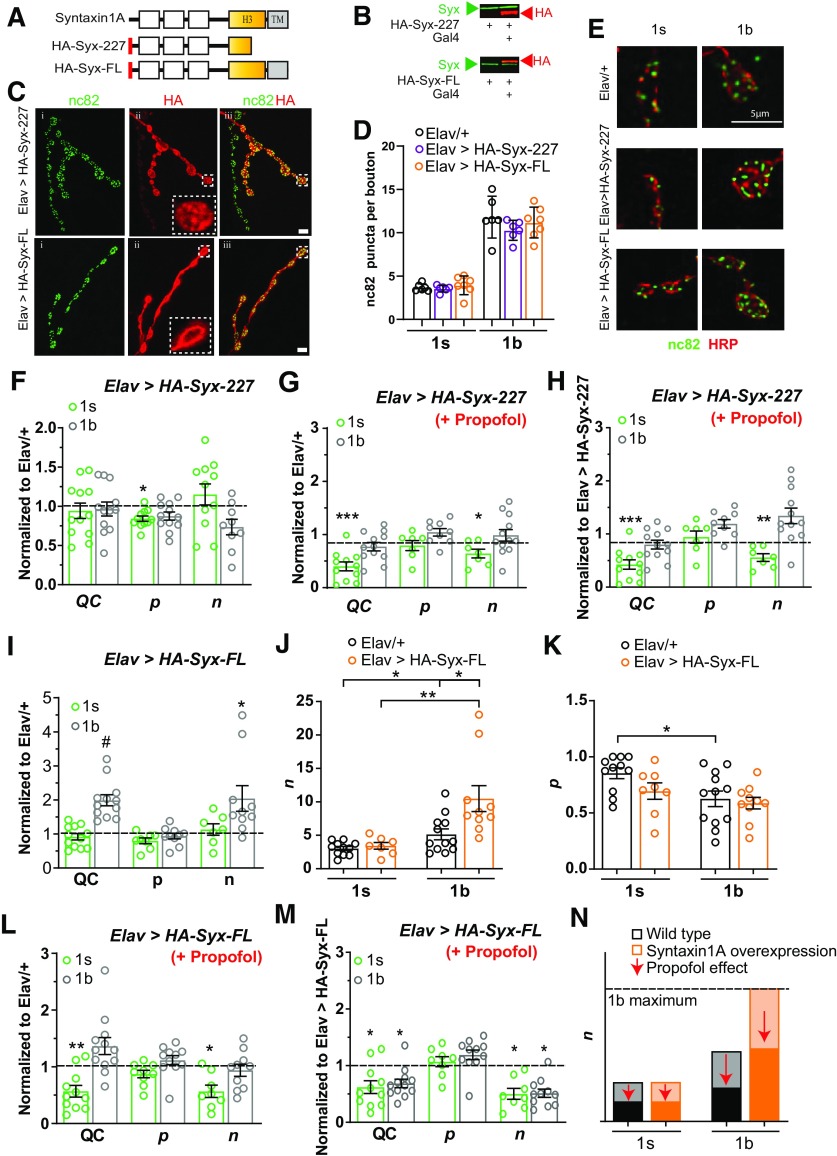
Syntaxin1A manipulations confer resistance to propofol by preserving or increasing the number of active release sites. ***A***, Schematic representing the wild-type syntaxin1A protein, an HA-tagged truncated syntaxin1A construct (*HA-Syx-227*), and an HA-tagged full-length syntaxin1A construct (*HA-Syx-FL*). H3, SNARE interaction domain; TM, transmembrane domain. ***B***, Coexpression of the truncated and full-length constructs in *Drosophila* shown by Western blot: endogenous wild-type syntaxin1A (green), *HA-Syx-227*, and *HA-Syx-FL* (red). ***C***, Immunolabeling of larval boutons in *HA-Syx-227* and *HA-Syx-FL*: left column, synaptic marker, nc82 (green); middle column, HA-labeling of coexpressed syntaxin1A (red, magnification of a single bouton is inset, showing localization of the coexpressed truncated syntaxin1A protein); right column, merge of nc82 and HA labels. Scale bar, 4 μm. ***D***, Average number of active zones (±SEM) in *Elav > HA-Syx-227* (*N* = 6) and *Elav > HA-Syx-FL* animals (*N* = 7) compared with *Elav/+* controls (*N* = 6), for 1s and 1b boutons. ***E***, Example boutons (labeled with HRP) and puncta (labeled with nc82) for the same strains as in ***D***. ***F***, QC, *p* and *n* for the two bouton types in *Elav > HA-Syx-227*, normalized to *Elav/+* (dashed line; Extended Data Fig. 4-1). ***G***, QC, *p* and *n* for the two bouton types in *Elav > HA-Syx-227 *+* *propofol, normalized to *Elav/+* (dashed line; Extended Data Fig. 4-2). ***H***, QC, *p* and *n* for the two bouton types in *Elav > HA-Syx-227 *+* *propofol, normalized to *HA-Syx-227* without propofol (dashed line; Extended Data Fig. 4-3). ***I***, QC, *p* and *n* (±SEM) for the two bouton types in *Elav > HA-Syx-FL*, normalized to *Elav/+* (dashed line; Extended Data Fig. 4-4). ***J***, Average number (±SEM) of estimated active release sites (*n*) in *Elav > HA-Syx-FL* animals, for 1b and 1s boutons. ***K***, Average estimated release probability (*p* ± SEM) in *Elav > HA-Syx-FL* animals, for 1b and 1s boutons. ***L***, QC, *p* and *n* (± SEM) for the two bouton types in *Elav > HA-Syx-FL* + propofol, normalized to *Elav/+* (dashed line; Extended Data Fig. 4-5). ***M***, QC, *p* and *n* (± SEM) for the two bouton types in *Elav > HA-Syx-FL* + propofol, normalized to *HA-Syx-FL* without propofol (dashed line; Extended Data Fig. 4-6). Individual data points in ***F–M*** are animals. *p <* *0.05; **p <* *0.01; ***p <* *0.001, ^#^*p *<* *0.0001, Student’s *t* test. ***N***, Summary schema of the opposing effects of syntaxin1A overexpression and propofol on the number of active release sites (*n*). Physiologic maxima for 1b boutons is indicated. Darker shading below arrows represents the proportional decrease in *n*, under propofol exposure.

Release sites require a variety of molecular infrastructure, including the target SNARE syntaxin1A (Südhof and Rizo, 2011), and components of the synaptic release machinery have been implicated in modulating general anesthesia phenotypes (Xie et al., 2013; van Swinderen and Kottler, 2014; Bademosi et al., 2018). Experiments in cultured neurosecretory cells have shown that the effects exerted by propofol on neurotransmitter release can be completely blocked by coexpressing a truncated syntaxin1A protein alongside endogenous syntaxin1A (Herring et al., 2011; Bademosi et al., 2018). Similar truncated syntaxin1A proteins have also been shown to block the effect of volatile anesthetics *in vivo* as well as *in vitro* (van Swinderen et al., 1999; Metz et al., 2007; Herring et al., 2009). We therefore investigated whether the coexpression of a truncated syntaxin1A protein in *Drosophila* larvae could block the specific effect of propofol that we have uncovered here, as this would confirm that preserving *n* is key to conferring resistance to the presynaptic effects of the drug. To test this, we expressed a similarly truncated syntaxin1A in *Drosophila* (HA-Syx-227; Fig. 4*A*), driven by the UAS/Gal4 system (Duffy, 2002). We inserted an HA tag onto the N terminus of the protein (Fig. 4*A*, red bar) to enable us to verify its expression in the wild-type syntaxin1A background. We confirmed that the truncated syntaxin1A protein is expressed in *Drosophila* larvae (Fig. 4*B*, top) and then examined its localization in motoneurons by colabeling with the active-zone marker *bruchpilot* (nc82; Wagh et al., 2006; Fig. 4*C*). Interestingly, although HA-Syx-227 was strongly expressed in 1b nerve terminals, expression levels in 1s boutons were low to nondetectable (Fig. 4*C*, top row). This differential expression pattern gave us an opportunity to compare the effects of propofol on manipulated versus “control” nerve terminals within the same animals. Importantly, coexpressing the truncated syntaxin1A protein did not change the average number of anatomic release sites (active zones) per bouton, compared with controls (Fig. 4*D*,*E*).

10.1523/ENEURO.0422-19.2020.f4-1 Figure 4-1 ***A–E***, Raw data of EJC amplitude (***A***), mEJC amplitude (***B***), QC (***C***), *p* (***D***), and *n* (***E***), used to generate the normalized data in Figure 3*F*. *p <* *0.05; **p <* *0.01; ***p <* *0.001; #*p *<* *0.0001; Student’s *t* test. Graphs show individual data points and the error bars show average ± SEM. Download Figure 4-1, EPS file.

10.1523/ENEURO.0422-19.2020.f4-2 Figure 4-2 ***A–E***, Raw data of EJC amplitude (***A***), mEJC amplitude (***B***), QC (***C***), *p* (***D***), and *n* (***E***), used to generate the normalized data in Figure 3*G*. *p <* *0.05; **p <* *0.01; ***p <* *0.001; #*p *<* *0.0001; Student’s *t* test. Graphs show individual data points and the error bars show average ± SEM. *Elav/+* data are the same as in Extended Data Figure 4-1. Download Figure 4-2, EPS file.

10.1523/ENEURO.0422-19.2020.f4-3 Figure 4-3 ***A–E***, Raw data of EJC amplitude (***A***), mEJC amplitude (***B***), QC (***C***), *p* (***D***), and *n* (***E***), used to generate the normalized data in Figure 3*H*. *p <* *0.05; **p <* *0.01; ***p <* *0.001; #*p *<* *0.0001; Student’s *t* test. Graphs show individual data points and the error bars show average ± SEM. *Elav>HA-Syx-227* data are the same as in Extended Data Figure 4-1. *Elav>HA-Syx-227* (+Propofol) data are the same as in Extended Data Figure 4-2. Download Figure 4-3, EPS file.

10.1523/ENEURO.0422-19.2020.f4-4 Figure 4-4 ***A–C***, Raw data of EJC amplitude (***A***), mEJC amplitude (***B***), and QC (***C***) used to generate the normalized data in Figure 3*I*. *p <* *0.05; **p <* *0.01; ***p <* *0.001; #*p *<* *0.0001; Student’s *t* test. Graphs show individual data points and the error bars show average ± SEM. Elav/+ data are the same as in Extended Data Figures 4-1 and 4-2. Download Figure 4-4, EPS file.

10.1523/ENEURO.0422-19.2020.f4-5 Figure 4-5 ***A–E***, Raw data of EJC amplitude (***A***), mEJC amplitude (***B***), QC (***C***), *p* (***D***), and *n* (***E***), used to generate the normalized data in Figure 3*L*. *p <* *0.05; **p <* *0.01; ***p <* *0.001; #*p *<* *0.0001; Student’s *t* test. Graphs show individual data points and the error bars show average ± SEM. *Elav/+* data are the same as in Extended Data Figure 4-4 and Figure 4, *J* and *K*. Download Figure 4-5, EPS file.

10.1523/ENEURO.0422-19.2020.f4-6 Figure 4-6 ***A–E***, Raw data of EJC amplitude (***A***), mEJC amplitude (***B***), QC (***C***), *p* (***D***), and *n* (***E***), used to generate the normalized data in Figure 3*M*. *p <* *0.05; **p <* *0.01; ***p <* *0.001; #*p *<* *0.0001; Student’s *t* test. Graphs show individual data points, and the error bars show the average ± SEM. Elav > HA-Syx-FL data are the same as in Extended Data Figure 4-4, and *Elav > HA-Syx-FL* (+Propofol) data are the same as in Extended Data Figure 4-5. Download Figure 4-6, EPS file.

In the absence of propofol, coexpression of truncated syntaxin1A revealed no significant change in baseline QC or *n* compared with control animals (*Elav>Syx-227* normalized to *Elav/+*), for 1b or 1s boutons (Fig. 4*F* and Extended Data Fig. 4-1*A–E*). We did note, however, a decrease in ECJ and mEJC amplitudes in 1s boutons, as well as a decrease in *p* (Extended Data Fig. 4-1*A*,*B*,*E*), which suggests a residual transgenic effect in these smaller boutons. In the presence of propofol, QC and *n* were decreased only at the 1s boutons, whereas QC and *n* remained at control levels in the 1b boutons that strongly expressed the truncated syntaxin1A protein (Fig. 4*G* and Extended Data Fig. 4-2*A–E*). We next assessed propofol effects within the same *Elav>Syx-227* strain. Consistent with previous studies in cell culture (Herring et al., 2011; Bademosi et al., 2018), coexpression of the truncated syntaxin1A construct completely blocked the effect of propofol on neurotransmitter release from 1b boutons expressing the mutant protein (Fig. 4*H* and Extended Data Fig. 4-3*A–E*), whereas in control 1s boutons (in the same animals) the number of active release sites was decreased by half (Fig. 4*H*).

If propofol decreases the number of active release sites, then increasing this number should reset the effect of the drug and thereby confer a level of resistance. We therefore sought a way to artificially increase the number of active release sites in nerve terminals. We hypothesized that providing synapses with a surplus of wild-type syntaxin1A might increase the number of active release sites per bouton. To test this, we expressed a full-length (FL) wild-type syntaxin1A in *Drosophila* (HA-Syx-FL; Fig. 4*A*). We again inserted an HA tag onto the N terminus of the protein (Fig. 4*A*, red bar) to enable us to track its expression in an endogenous syntaxin1A background. We verified that the transgenic wild-type syntaxin1A was indeed expressed in *Drosophila* larvae (Fig. 4*B*, bottom), and then observed that the wild-type construct was expressed in both synaptic bouton types in motor nerve terminals (Fig. 4*C*, bottom row). Interestingly, overexpressing wild-type syntaxin1A did not increase the average number of active zones in 1s or 1b boutons, as measured by counting nc82 puncta in *Elav>HA-Syx-FL* animals, compared with *Elav/+* controls; the syntaxin1A overexpressing line exhibited ∼4 and 10 puncta per 1s and 1b boutons, respectively (Fig. 4*D*,*E*).

We next assessed the effect of syntaxin1A overexpression on synaptic physiology in the absence of propofol, in *Elav>HA-Syx-FL* animals compared with *Elav/+* controls. We found that overexpression of syntaxin1A caused QC to be increased at 1b but not at 1s boutons (Fig. 4*I* and Extended Data Fig. 4-4*A–C*). The increase in 1b QC resulted from an increase in *n* and not in *p* (Fig. 4*I–K*). *n* was increased to ∼10 in 1b boutons (Fig. 4*J*), matching the average number of active zones in these boutons (Figs. 2*C*, 4*D*). In 1s boutons, *n* remained the same, at approximately four per bouton (Fig. 4*J*), matching the average number of active zones in those boutons (Figs. 2*C*, 4*D*).

Syntaxin1A overexpression therefore recruits additional active release sites at 1b boutons, but not 1s boutons. Following our hypothesis, this genetic manipulation should reset the effect of propofol in 1b boutons specifically. This is indeed what we observed: as 1b boutons in syntaxin1A-overexpressing (*Elav<HA-Syx-FL*) animals had an increased *n* value (Fig. 4*J*), the net effect of propofol in this strain was to return *n* (and thus QC) to control (*Elav/+*) levels (Fig. 4*L*, dashed line), whereas the decrease in *n* (and QC) at 1s boutons was below control levels (Fig. 4*L* and Extended Data Fig. 4-5*A–E*).

Despite this differential effect of syntaxin1A overexpression on 1b and 1s boutons, we found that propofol still decreased QC at both bouton types by similar proportions, in animals overexpressing syntaxin1A (Fig. 4*M* and Extended Data Fig. 4-6*A–C*). This decrease in QC was due to equivalent effects on *n* at both types of synapse, with no effect on *p* (Fig. 4*M* and Extended Data Fig. 4-6*D*,*E*). Increasing the number of active release sites in 1b boutons therefore does not change the net effect of propofol, which is to decrease *n* by about half. Rather, 1b boutons are relatively less impacted by the drug in *Elav<HA-Syx-FL* animals because of an artificially higher *n* baseline (Fig. 4*J*). In conclusion, propofol has a proportionally equivalent effect impairing neurotransmitter release from either type of synapse, but a capacity to increase the number of active release sites in 1b boutons (Figs. 2*I*, 4*J*) counteracts its effect at these synapses specifically (Fig. 4*N*).

## Discussion

In this work, we have provided evidence that a clinically relevant concentration of the intravenous general anesthetic propofol impairs excitatory neurotransmission by decreasing (by half) the availability of active release sites at presynaptic terminals. Our findings are intriguing because they point to a likely mechanism whereby drugs such as propofol might be impairing presynaptic release: not by decreasing the release probability of already primed (i.e., active) release sites, but instead by altering the ratio between active and inactive sites. Most presynaptic terminals involve a dynamic balance of active and inactive release sites, and this balance can vary across synapse types (Neher and Brose, 2018). By recording from individual boutons at the *Drosophila* NMJ, we have shown that knowing the *n* value of a bouton is key to understanding the effect of propofol on neurotransmission. We show that even within similar bouton types *n* can vary widely, so to capture the effect of propofol on *n* requires a bouton-by-bouton assessment of the number of active release sites that are available. This would have been missed by simply averaging all bouton recordings or by only measuring combined 1s and 1b potentials recorded from postsynaptic tissue. Similarly, previous studies investigating presynaptic effects of propofol at the frog NMJ (Leite et al., 2011) or in rat cortical cultures (Kitamura et al., 2003) did not acquire physiological data allowing estimates of release site numbers, as we have done here. It is interesting to note, however, that propofol exposure increases quantal size in the both the fly (Extended Data Fig. 1-1*B*) and frog (Leite et al., 2011) NMJ.

Earlier work on the vertebrate NMJ showed that opioids and adenosine also inhibit neurotransmission by decreasing *n* (Bennett et al., 1991; Lavidis, 1995). These compounds activate presynaptic autoreceptors, which then initiate a cascade of events leading to decreased neurotransmitter release via an estimated decrease in *n*. As in those earlier vertebrate studies, our estimates of *n* in fly larval boutons are purely electrophysiological. However, our electrophysiological estimates of *n* maxima match well with anatomic counts of T-bar numbers or BRP puncta in fly larval boutons, providing confidence that our estimates of the effect of propofol on *n* are accurate. There are currently no reliable imaging or immunochemical techniques for disambiguating active from inactive release sites, although methods aimed at tracking the molecular dynamics and conformation of key vesicular priming proteins such as *munc13* (Dittman, 2019) provide a promising future research direction to verify our electrophysiological observations.

One interpretation of our results is that propofol converts a proportion of active sites with a high release probability to inactive sites with low release probability. Alternatively, propofol could inhibit inactive sites from converting to active sites. Our data do not reveal the directionality of this effect, only that propofol increases the number of inactive sites without affecting the release probability of remaining active sites. Thus, release sites unaffected by propofol presumably remain fully functional (rather than half-functional, for example), as evidenced by our finding that release probability at the fly NMJ is not impaired by propofol. In previous studies, we have suggested that propofol may act on components of the synaptic release machinery prior to SNARE formation (Bademosi et al., 2018), which is consistent with our current finding that *n* is affected rather than *p*. Additionally, we have shown that the resistance-inducing truncated syntaxin1A protein is not incorporated into the SNARE complex (Troup et al., 2019), which again suggests a role prior to SNARE formation. One possibility that we have raised before (Bademosi et al., 2018) is that propofol inhibits a step in the formation of functional release sites, thereby creating “traffic jams” of inactive sites that fail to become fully functional. A level of resistance to propofol is thus afforded at some boutons, depending on the availability of additional inactive release sites, which could be recruited to become active, depending on the physiological demands of a neuron. Thus, overexpressing syntaxin1A in 1b boutons maintains glutamate release near wild-type levels in the presence of propofol because these larger boutons have additional release sites that can be recruited to become high-release probability (thus active) sites. On the other hand, coexpressing a truncated syntaxin1A in 1b boutons preserves *n* and thus QC from the effects of propofol specifically in these boutons, while *n* is again halved in unprotected 1s boutons in the same tissue. Together, these genetic manipulations provide compelling evidence that, alongside its known effects on postsynaptic receptors and ion channels (Franks, 2008; Hemmings et al., 2019), propofol impairs neurotransmission by decreasing the availability of active release sites. Genetic manipulations of different components of the SNARE assembly and priming processes should clarify whether propofol renders active sites inactive, or whether it prevents inactive sites from becoming active.

Although synaptic release mechanisms are extremely conserved across animals (Südhof and Rizo, 2011), not all presynapses are the same. As we have shown here, even synapses releasing the same neurotransmitter (glutamate) onto the same muscle can be physiologically different. 1s boutons have a higher release probability (Figs. 2*H*, 3*B*), and all of their release sites are active (Fig. 2*J*,*K*), whereas 1b boutons are weaker (Extended Data Fig. 1-1*A*) but have a capacity to recruit more release sites (Figs. 2*I*, 4*J*). Yet, both types of boutons are impacted in a similar way by propofol, with the drug decreasing the number of active release sites by half. If propofol impairs neurotransmitter release by proportionally decreasing *n* by half, as we show here, then smaller boutons (e.g., 1s in our study) could be more vulnerable to the presynaptic effects of the drug, and this would especially be the case for synapses with only one active zone, which probably describes most excitatory synapses in the brain (Schikorski and Stevens, 1997). Extrapolating to humans, this may explain why some circuits are more affected than others by surgical concentrations of general anesthetics such as propofol (Kim et al., 2007). Future work should reveal whether the presynaptic effect that we have uncovered for propofol extends to other types of synapses (e.g., inhibitory) and neurotransmitter systems, and whether this effect may be a common presynaptic mechanism of other general anesthetics.

## Synthesis

Reviewing Editor: Niraj Desai, NINDS Decisions are customarily a result of the Reviewing Editor and the peer reviewers coming together and discussing their recommendations until a consensus is reached. When revisions are invited, a fact-based synthesis statement explaining their decision and outlining what is needed to prepare a revision will be listed below. The following reviewer(s) agreed to reveal their identity: Andrew Hudson. Note: If this manuscript was transferred from JNeurosci and a decision was made to accept the manuscript without peer review, a brief statement to this effect will instead be what is listed below.

The reviewers and reviewing editor were all impressed by the carefulness of this study and are (largely) convinced of its conclusions. Even so, there are several places where the study might be strengthened, either by including additional data, further analysis of existing data, or revision of the text to address outstanding questions.

1. ACTIVE SITES OF 1s BOUTONS

In Figure 2, the authors show that increasing the extracellular calcium concentration from 1 mM to 1.5 mM had no effect on 1s boutons but increased n and quantal content (QC) in 1b boutons. Based on this result, they conclude that, at physiological calcium concentrations, all release sites are active in 1s boutons. But to make this argument, it is important to show that lowering the calcium concentration - perhaps to 0.5 mM - will reduce n and QC at 1s boutons.

2. PROPOFOL CONCENTRATION

One of the main findings of this manuscript is that propofol (3 μM) reduces the number of active release sites by half. This is observed in 1s and 1b boutons in control animals (Fig. 1) and is also observed in 1b boutons overexpressing wild-type Syntaxin 1A (Fig. 4L). Why does propofol decrease the number of release sites by half? Is this effect specific to the concentration of propofol used? If so, would a higher concentration of propofol block all active release sites? It would be extremely useful if the authors can include an explanation for this in the manuscript.

A direct way of testing this question would be to measure the effect of a higher concentration of propofol on n in 1b boutons in Elav>HA-Syx-FL animals. It is possible that even with the increased concentration of propofol, the effect on n will be the same (i.e. the number of release sites will be reduced by half). Alternatively, there could be a dose-dependent effect of propofol on the presynaptic release sites. Either way, this experiment would provide important insights into the presynaptic effects of propofol.

3. SYNTAXIN 1A & 1s BOUTONS

The truncated synataxin 1A protein is not expressed in 1s boutons. However, the EJC amplitudes, mEJC amplitudes, and release probability at 1s boutons are all reduced in Elav>HA-Syx-277 animals (compared to controls). The authors should mention these results in the text and comment on the possible reasons for this phenotype.

4. VARIABILITY IN QUANTAL CONTENT

It is striking that, in Fig. 1D, the variability in QC does not seem to decrease with the application of propofol relative to the control analog; that is, the data range for each neuron type is almost the same for the high and low QC conditions, just shifted up or down. One might have expected that, since QC = np, the distribution of observed QCs would resemble a binomial distribution convolved with some release probability distribution, and hence would have decreased in range as n decreased. Is this reasoning wrong or is this possibly due to varying drug concentrations over the duration of the experiment? Or is there something else at play?

5. mEJC PROPERTIES

The authors should be able to extract the numbers for mEJC frequency, kinetics and charge from their existing recordings (data in Fig. 1-1B). It would be useful if the authors could show the effects of propofol on these parameters in the manuscript.

6. RECORDING STABILITY

How stable are these recordings over time? The drug is flowed over the boutons before the patch is applied and not once the measurements are conducted. This raises the question of how long a recording session takes and whether the propofol concentration within the bouton would be stable over this interval.

7. MISSING REFERENCE

The authors do not cite the 2011 paper by Leite et al., which assessed the impact of propofol on synaptic release at the neuromuscular junction of the frog with the fluorescent dye FM1-43. Those authors reported increased vesicle release at low doses of propofol (10-25 micromol/L) that they suspected involved N-type calcium channels, as well as a decrease in sodium-dependent exocytosis at higher concentrations of propofol (50-200 micromol/L). Given that this is one of the most directly relevant studies of propofol on synaptic vesicle cycling at an NMJ (albeit in a vertebrate), I would think the authors would want to address it.

8. DETAILS OF METHODS

In the Methods section, the authors have provided most methodological details and the rationale for their experimental design. However, a few things are missing: (1) details of the stimulating electrode, stimulation protocol (frequency), and stimulation intensity; (2) the specific criteria by which the authors classified bouton types into 1s and 1b; and (3) data acquisition rates used for focal macropatch recordings.

9. MINOR COMMENTS

(A) The authors should mention “fly neuromuscular junction” or “fly motor neurons” in the title of the manuscript. (B) Page 3, line 48, missing “of” after “counteracting the effect”. (C) Page 17, line 374, missing “to” after “in order”.

## Author Response

eNeuro eN-TNWR-0422-19X: Reply to Reviewers

1) In Figure 2, the authors show that increasing the extracellular calcium concentration from 1 mM to 1.5 mM had no effect on 1s boutons but increased n and quantal content (QC) in 1b boutons. Based on this result, they conclude that, at physiological calcium concentrations, all release sites are active in 1s boutons. But to make this argument, it is important to show that lowering the calcium concentration - perhaps to 0.5 mM - will reduce n and QC at 1s boutons.

The reviewer suggests that to show that all 1s release sites are participating at the physiological [Ca2+]o of 1.5 mM, we might need to show that n and QC are reduced when [Ca2+]o is lowered to 0.5 mM. In this manuscript, we showed that all release sites within both 1b and 1s boutons, and not just within 1s boutons, are active at [Ca2+]o= 1.5 mM by taking an alternative approach, which we believe is more direct than that suggested by the reviewer. To show that all the release sites are active within a bouton at [Ca2+]o= 1.5 mM, we tested the hypothesis that n should equate to the number of anatomical release sites within a bouton (Fig. 2c). At 1b boutons, at [Ca2+]o= 1.0 mM, n is lower than the number anatomical release sites within a bouton (Fig. 2i, k) of approximately 8 (Fig. 2c). When the [Ca2+]o was raised to 1.5 mM, n equated to the number of anatomical release sites (Fig. 2i, k), and QC was also increased (Fig. 2g). At [Ca2+]o= 1.5 mM, all release sites were therefore active within 1b boutons. At 1s boutons, at [Ca2+]o of 1mM, n had already reached its upper limit (Fig. 2i, k), equating to the number of anatomical release sites of approximately 4 (Fig. 2c). When the [Ca2+]o was raised to 1.5 mM, we anticipated not observing a further increase in n since the upper limit had already been reached at the lower [Ca2+]o. This was indeed the case (Fig. 2i, k). Together, these results indicate that all the release sites within 1b and 1s boutons are active at [Ca2+]o= 1.5 mM.

At chemical synapses, a decrease in [Ca2+]o would produce a decrease in neurotransmitter release. Since QC is positively correlated with n at both 1b and 1s boutons (Fig. 3d), a lowering of [Ca2+]o would decrease QC through a decrease in n. Since 1s boutons are releasing at their their upper limit at [Ca2+]o= 1.0 mM, lowering the [Ca2+]o below that level would therefore decrease QC through a decrease in n. We hope that this is a sufficient explanation to convince the reviewer and readers.

2) One of the main findings of this manuscript is that propofol (3 μM) reduces the number of active release sites by half. This is observed in 1s and 1b boutons in control animals (Fig. 1) and is also observed in 1b boutons overexpressing wild-type Syntaxin 1A (Fig. 4L). Why does propofol decrease the number of release sites by half? Is this effect specific to the concentration of propofol used? If so, would a higher concentration of propofol block all active release sites? It would be extremely useful if the authors can include an explanation for this in the manuscript.

A direct way of testing this question would be to measure the effect of a higher concentration of propofol on n in 1b boutons in Elav>HA-Syx-FL animals. It is possible that even with the increased concentration of propofol, the effect on n will be the same (i.e. the number of release sites will be reduced by half). Alternatively, there could be a dose-dependent effect of propofol on the presynaptic release sites. Either way, this experiment would provide important insights into the presynaptic effects of propofol.

This is an interesting question: does propofol have a similar effect on QC (and n or p) at higher concentrations? We went ahead and tested this as proposed by the reviewer, by examining effects of 10x the propofol concentration (30μM) on 1b boutons in Elav>HA-Syx-FL animals (Figure R1). Although we found decreased QC at this higher concentration (QC at 30 μM propofol = 1.01±0.45 vs 3.75±0.41 at 3 μM propofol; n=9 and 12, respectively), we do not think that these data are appropriate for making any conclusions about the effect on the number of active release sites (n). This is because of the high number of failures observed with 30μM propofol (52.6±14.3 at 30 μM propofol vs 0.04±0.04 at 3 μM propofol; n=9 and 12, respectively). This indicates that at higher concentrations, additional factors are being compromised by propofol, such as the prevention of motoneuron action potentials from invading the nerve terminals to evoke release. We have decided not to include the data in Figure R1 in our paper as it is peripheral to our focus on quantal content effects at clinically relevant drug concentrations. However, we have included some additional text in the Methods explaining about failure rates at higher drug doses (lines 281-285), as it is important to know that there about potentially confounding effects at supra-clinical drug concentrations; failures obviously affect neurotransmission at higher concentrations, but this is misleading with regard to effects on quantal content and synaptic release, the focus of this study.

3) The truncated syntaxin 1A protein is not expressed in 1s boutons. However, the EJC amplitudes, mEJC amplitudes, and release probability at 1s boutons are all reduced in Elav>HA-Syx-277 animals (compared to controls). The authors should mention these results in the text and comment on the possible reasons for this phenotype.

The reviewer is referring to the data shown in supplemental Figure 4-1 (panels A, B, and E), associated with Figure 4. Indeed, these metrics are decreased in 1s boutons, compared to controls in the absence of propofol, and this is not evident when only quantal content is presented in the main figure. This is a keen observation. Our only explanation is that the truncated syntaxin1A is expressed at low levels in 1s boutons, which appears to be the case if one looks closely at Fig. 4C, upper middle panel. It is possible that such a low level of expression is insufficient to be protective against propofol (as in 1b boutons), but enough to compromise native release from 1s boutons a little. We have now mentioned these observations in the main text (lines 492-494), and adjusted our language correspondingly about 1s expression levels.

4) It is striking that, in Fig. 1D, the variability in QC does not seem to decrease with the application of propofol relative to the control analog; that is, the data range for each neuron type is almost the same for the high and low QC conditions, just shifted up or down. One might have expected that, since QC = np, the distribution of observed QCs would resemble a binomial distribution convolved with some release probability distribution, and hence would have decreased in range as n decreased. Is this reasoning wrong or is this possibly due to varying drug concentrations over the duration of the experiment? Or is there something else at play?

The reviewer observes that the variability in QC was not decreased in the presence of the drugs- especially in the presence of propofol- and sought an explanation for this observation. At both 1b and 1s boutons, the decrease in QC (Fig. 1D) caused by propofol resulted from a decrease in n and not in p (Fig. 3C). On average, half of the active release sites are vulnerable to propofol action whereas the remaining half are resistant to propofol action at the administered [Propofol]= 3 μM. Propofol does not therefore completely inactivate all release sites within a bouton (Fig. 3A); otherwise the variation in QC would be decreased since neurotransmitter release would be muted from some boutons. Because 50% of release sites remain unaffected by propofol action, p is never reduced to zero at either 1b or 1s boutons. Our finding that p is unaffected by propofol at the fly NMJ is consistent with our previous work suggesting that propofol acts prior to full SNARE formation. We have now added some lines in the discussion clarifying this view and conclusion (lines 580-582).

5) The authors should be able to extract the numbers for mEJC frequency, kinetics and charge from their existing recordings (data in Fig. 1-1B). It would be useful if the authors could show the effects of propofol on these parameters in the manuscript.

The mEJC frequency and kinetics are now provided in Fig. 1-1D-G. The mEJC charge could not be ascertained because these currents were not obtained through patch clamp recordings, but through extracellular recordings (Karunanithi et al, 2018). We now mention these additional metrics in the results (lines 380-388, pointing to the corresponding figure panels in Fig. 1-1.

Figure 1-1: mEJC frequency and kinetics. A, 1b and 1s mEJC frequencies recorded from single boutons in control and in drug-treated preparations. B, An exemplary mEJC trace (red) showing the measure of rise time and the single exponential fit to the decay phase (black) from which the decay constant is derived. C-D, mEJC rise time (C) and decay constant (D). Significant differences are shown: *P<0.05; **P<0.01; Student's t-test. Graphs show individual data points and error bars show average + s.e.m.

Results: 1b and 1s mEJC frequencies were unaffected by either propofol or the analog (Fig 1-1D).

Change in mEJC rise time or decay constant (Fig. 1-1E) following drug treatment would indicate that postsynaptic receptor kinetics are affected by the drugs. Propofol did not affect either rise time or decay constant at either bouton type (Fig. 1.1F,G). The analog however prolonged both those parameters at 1b boutons (Fig. 1.1F,G).

Methods: mEJC rise time and decay constant were measured using LabChart Pro (v8.1.5, ADInstruments) software. As previously, rise times were measured manually by using the two cursors placed at the base and the peak of each mEJC (Karunanithi et al., 1999). The mEJC decay constant was measured using the Peak Analysis subroutine, using the ‘General-unstimulated’ analysis type and ‘simple threshold’ peak detection setting. ‘Tau’ was selected in the Table options to derive the decay constant. These additional method have been added to our Methods section (lines 256-261).

6) How stable are these recordings over time? The drug is flowed over the boutons before the patch is applied and not once the measurements are conducted. This raises the question of how long a recording session takes and whether the propofol concentration within the bouton would be stable over this interval.

In each experiment, recordings would take approximately 3.33 mins for EJCs and 5-10 mins for mEJCs. In recordings, the drug was present in the perfusate before and during the recordings and the perfusate was perfused through the recording chamber at a rate of 1-2 mL per min for over 30 - 40 mins (Karunanithi et al., 2018). The focal macropatch electrode was also immersed in the perfusate before the recording session, ensuring that the drug concentration within the lumen of the electrode was the same as that outside of it. We found that it was necessary to pre-expose the preparation to propofol in order to detect an effect of the drug on neurotransmission, probably because drug access at the level of the recorded bouton is impeded once a light seal has been achieved. However, because the rim of the focal macropatch electrode does not form a tight seal with the muscle membrane (Karunanithi et al., 2002), following equilibration the propofol-containing perfusate is probably able to cross into the lumen of the electrode, flow across the boutons, and then into the outside of the lumen. There is slow exchange of perfusate within the lumen of the electrode and this contributes to the propofol concentration remaining stable throughout the recording session. The alternative approach of perfusing propofol solution only after patching the bouton was impractical and led to variable results, as it was impossible to ascertain that propofol had reached the recording tissue in question. We have now added some lines to the Methods clarifying our recording rationale (lines 265-268, 274-281).

7) The authors do not cite the 2011 paper by Leite et al., which assessed the impact of propofol on synaptic release at the neuromuscular junction of the frog with the fluorescent dye FM1-43. Those authors reported increased vesicle release at low doses of propofol (10-25 micromol/L) that they suspected involved N-type calcium channels, as well as a decrease in sodium-dependent exocytosis at higher concentrations of propofol (50-200 micromol/L). Given that this is one of the most directly relevant studies of propofol on synaptic vesicle cycling at an NMJ (albeit in a vertebrate), I would think the authors would want to address it.

Thank you for the Leite et (2011) reference, we now cite this in the discussion (lines 559-563, along with another paper that examined the effect of propofol on rat cortical cultures (Kitamura et al, 2003). We believe the effects we report in our study have been missed in previous studies because they were not specifically estimating n, the number of active release sites. We show that release probability is unaffected by propofol, so in other preparations it is likely that presynaptic propofol effects would have gone undetected. Interestingly, as in the Leite 2011 study, we also found that propofol increased mEJC amplitudes (see Figure 1-1B). The other effects presented in the Leite 2011 study are supra-clinical, so difficult to relate to our work. All of the rat cortical culture work (Kitamura 2003) is spontaneous release, not evoked, so impossible to derive any estimate of active release site numbers.

8) In the Methods section, the authors have provided most methodological details and the rationale for their experimental design. However, a few things are missing: (1) details of the stimulating electrode, stimulation protocol (frequency), and stimulation intensity; (2) the specific criteria by which the authors classified bouton types into 1s and 1b; and (3) data acquisition rates used for focal macropatch recordings.

We have now added these additional details to the Methods section (lines 222-226, 234-237). Briefly, the stimulating electrode (of lumen diameter of approximately 10-12 μm), filled with perfusate, was used to gently suck the cut end of the segmental nerve and was stimulated at a frequency of 1 Hz at supramaximal strengths. 200 of the resulting EJCs were recorded. 25-50 mEJCs were also recorded in each experiment. Data was digitised at an acquisition rate of 40 KHz. We can readily differentiate between the larger 1b and smaller 1s boutons under Nomarski optics (Kurdyak et al, 1994; Karunanithi et al., 2018). 1b nerve terminals are composed of larger and more variable sized boutons (3-5 μm in diameter) than are 1s nerve terminals (boutons of ~3 μm in diameter) (Kurdyak et al., 1994; Atwood et al., 1993). Well-isolated 1b or 1s boutons were selected for recordings.

9) (A) The authors should mention “fly neuromuscular junction” or “fly motor neurons” in the title of the manuscript. (B) Page 3, line 48, missing “of” after “counteracting the effect”. (C) Page 17, line 374, missing “to” after “in order”.

(A) We have changed the title as requested. The new title is “Proportional downscaling of glutamatergic release sites by the general anesthetic propofol at Drosophila motor nerve terminals.

(B) Corrected

(C) Corrected
